# Spatial and temporal variability of bacterial communities within a combined sewer system

**DOI:** 10.1002/mbo3.356

**Published:** 2016-04-10

**Authors:** Henriette Stokbro Jensen, Raju Sekar, Will J. Shepherd, Andrew M. Osborn, Simon Tait, Catherine A. Biggs

**Affiliations:** ^1^ Department of Chemical and Biological EngineeringUniversity of SheffieldSheffieldUK; ^2^ Department of Biological SciencesXi'an Jiaotong‐Liverpool UniversitySuzhouChina; ^3^ Department of Civil and Structural EngineeringUniversity of SheffieldSheffieldUK; ^4^ School of ScienceRMIT UniversityBundooraVictoriaAustralia

**Keywords:** Bacteria, environmental biofilms, in‐sewer processes, sewer systems, T‐RFLP analysis, wastewater

## Abstract

This study describes the temporal and spatial variability of bacterial communities within a combined sewer system in England. Sampling was conducted over 9 months in a sewer system with intensive monitoring of hydraulic conditions. The bacterial communities were characterized by 16S rRNA gene‐targeted terminal restriction fragment length polymorphism analysis. These data were related to the hydraulic data as well as the sample type, location, and time. Temporal and spatial variation was observed between and within wastewater communities and biofilm communities. The bacterial communities in biofilm were distinctly different from the communities in wastewater and exhibited greater spatial variation, while the wastewater communities exhibited variability between different months of sampling. This study highlights the variation of bacterial communities between biofilm and wastewater, and has shown both spatial and temporal variations in bacterial communities in combined sewers. The temporal variation is of interest for in‐sewer processes, for example, sewer odor generation, as field measurements for these processes are often carried out over short durations and may therefore not capture the influence of this temporal variation of the bacterial communities.

## Introduction

Combined sewers are a key part of the urban infrastructure and play a vital role in safeguarding public health and reducing water pollution in the environment. Sewage released into the urban environment can potentially expose people to waterborne pathogens, and sewer overflows impact upon the natural aquatic environment as well as producing esthetic pollution including noxious odors. During the transport of wastewater within a combined sewer network, it undergoes physicochemical and biological changes (Hvitved‐Jacobsen 2002). Not only can these changes be caused by variable system inputs, such as wastewater and rainfall, but are also dependent on biological processes such as the degradation of organic matter and both the production and degradation of hydrogen sulfide (e.g., Hvitved‐Jacobsen 2002; Rudelle et al. 2011). Biological processes and the formation of biofilms on pipe walls have also been shown to influence the stability of in‐sewer sediment deposits (Schellart et al. 2005; Sakrabani et al. 2009) and change the roughness of the pipe surfaces, and therefore influence the hydraulic performance of the sewer pipes (Guzman et al. 2007). Currently, the performance of sewers is generally evaluated based on their hydraulic conditions, such as risk of sewer flooding; however, wastewater quality models are also increasingly being applied to describe changing physicochemical conditions with respect to the composition of the wastewater within sewer networks (Hvitved‐Jacobsen 2002; Jiang et al. 2009; Vollertsen et al. 2011).

Previous field observations have shown that wastewater composition in combined sewers can have a high degree of variability. The studies in combined sewers have mainly been carried out in Western Europe. For example, Gudjonsson et al. (2002), studying oxygen profiles measured over 24 h in a sewer network, showed that the oxygen concentration in the wastewater varied between 5 and 0 mg/L throughout a 24‐h period, occasionally decreasing by over 3 mg/L within an hour. Vollertsen et al. (2005) showed large spatial variability in chemical oxygen demand (COD) fractionation of the organic matter in 109 wastewater samples collected from five different in‐sewer locations in north Denmark. Measurements of biological oxygen demand (BOD5) in the inlet of a wastewater treatment plant by Evans et al. (2010) implied temporal variability in the inlet wastewater composition; however, changes in the microbial communities that are present within the supplying sewer network have seldom been investigated. The variable conditions in sewers, whether chemical or physical, can affect microbial communities in several ways. Biggs et al. (2011) showed that temperature affects the organic degradation processes in sewer sediments, changing not just the degradation rate of different organic substrates but also which organic substrates the microbial communities in the sewer degrade.

Biotransformation processes in the sewers are dependent on both the wastewater composition and the biofilm microbial communities, and in turn the microbial biofilm composition and activity are dependent on local environmental conditions within the pipe. Telgmann et al. (2004) investigated biofilm detachment under controlled laboratory conditions and found that flow conditions and shear stress had a significant effect on biofilm sloughing, structure, and strength. Moreover, Rochex et al. (2008) showed that increasing shear stress decreased species richness in a biofilm cultivated in a conical Couette–Taylor reactor inoculated with wastewater from a paper production plant. Ebrahimi et al. (2005) used a two‐dimensional mass balance model to illustrate the possibility of substrate availability as a contributing factor in determining biofilm growth patterns.

When assessing potential problems that are related to wastewater quality, for example, odor and corrosion problems, the understanding of microbial activity in both wastewater and biofilms and their interactions becomes important. This is because the formation of any problematic compounds is affected by both the organic matter transformation in the wastewater as well as the sulfur cycle in the sewer. The conceptual models describing the in‐sewer processes of organic matter transformation and the sulfur cycle rely on parameters describing the microbial activity of these different processes. Due to the high natural variation in concentrations and flows in sewer systems, some understanding of the statistical variability for these parameters is also required. However, it is not clear how this variability relates to spatial and temporal changes in microbial communities over longer timescales within a real sewer system. The aim of this study was therefore to investigate the spatial and temporal effects of local environmental conditions in a combined sewer network on the in‐sewer microbial communities in both the wastewater and sewer wall biofilms. The study was carried out in a heavily instrumented sewer network, so data on the prevailing hydraulic conditions were readily available. The hydraulic data had also been collected continuously at a high frequency for many months so short‐term and seasonal effects could be examined.

## Materials and Methods

**Figure 1:**
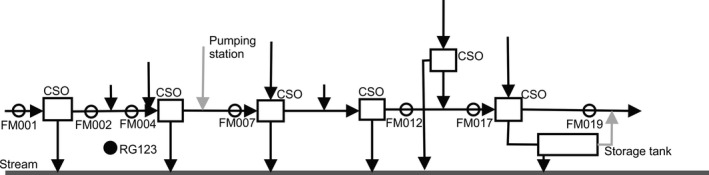
Schematic of the section of the sewer network where the samples were collected with the marked locations of the flow monitors (FM) in the intercepting sewer. Combined sewer overflows are marked with “CSO,” and RG123 denote the location of the rain gauge. Samples for bacterial community analysis were collected from the locations of flow meters, FM004, FM012, and FM019.

The study was carried out on an upstream section of a sewer system in the north of England with a contributing population of approximately 4370 and a contributing area of around 126 ha. The study area consists predominately of combined sewers with approximately 21 km of sewer pipes. The catchment is steep, with a median pipe slope for the sewer pipes of 0.0383 m/m and a maximum land slope of 0.128 m/m. The studied sewer network is arranged around an intercepting sewer that runs in a west–east direction, with numerous smaller sewer pipes draining residential areas running generally in a north–south direction. The network also has six combined sewer overflows (CSO) which release excess sewage into a small watercourse when the sewer network is overloaded during intense rainfall events; five of these are located on the main intercepting sewer (Fig. 1). The study area is part of a larger intensively monitored area which include 25 acoustic Doppler flow monitors located within the sewer network. These are used to collect high‐frequency (2 min) data on depth and velocity, with flow being calculated from these and the pipe dimensions. Of these flow monitors, seven were placed on the intercepting sewer (Shepherd et al. 2010). The catchment was selected for the study of bacterial community changes due to this availability of a long‐term extensive hydraulic dataset and the fact that there were no appreciable industrial input sources. The main intercepting sewer was identified as the most interesting sampling location. The general configuration of the system is outlined in Figure 1.

### Flow analysis for identification of sampling locations

The sites for the sampling of material for bacterial community analysis were chosen based on an analysis of the flow data from the seven flow monitors (FM001, FM002, FM004, FM007, FM012, FM017, and FM019) located on the intercepting sewer, as well as excluding sites where the access manhole was located in the main road as this would limit safe accessibility. Table 1 shows the period of available data from each of the seven chosen sampling sites. In addition to the flow monitors, there was one rain gauge placed within the catchment and located under 50 m from the closest flow monitor (FM002) and within 850 m of the furthest flow monitor (FM019). The availability of the rain data from a rain gauge located close to the study area is important when investigating combined sewers as these will convey rainwater as well as sewage during rain events. The data from this rain gauge were used to define days that had rain events within the catchment. A rain event was defined as more than 0.4 mm of rainfall in a day. All data collected on days where rain was registered from the rain gauge were initially eliminated from the dataset. In order to further ensure that the dataset did not contain wet weather flow, the data were further screened for days with high flow rates using Grubbs' (1950) outlier test, which was used to test if the day in the dataset with the highest flow was an outlier compared to the remaining dataset. In such a case it was removed as a day where the sewer flow was affected by rainfall, even though this was not seen on the rain gauge. The high flow days identified using the outlier test in most cases identified days following rain events and hence have an identifiable reason for having high flow due to rain runoff. Only a few additional days were identified which did not relate directly to measured rain events. However, as the rain gauge was placed in the southern part of the catchment and this slopes in the north to south direction, it is plausible that these few days with elevated flow correspond to rainfall in the elevated north of the catchment. Table 1 shows the number of days that are defined as dry weather days and was retained in the dataset for each station once the days with wet weather events and flow had been removed.

**Table 1:** Flow data collection period and number of dry days identified within this period

Location	Measuring period	Number of days with available data	Dry days
FM001	23 April 2008 to 13 August 2009	470	159 (34%)
FM002	23 April 2008 to 13 August 2009	470	148 (31%)
FM004	26 March 2008 to 13 August 2009	497	174 (35%)
FM007	26 March 2008 to 13 August 2009	497	153 (31%)
FM012	24 April 2008 to 13 August 2009	469	160 (34%)
FM017	25 March 2008 to 13 August 2009	498	162 (33%)
FM019	25 March 2008 to 6 July 2009	461	130 (28%)

**Figure 2:**
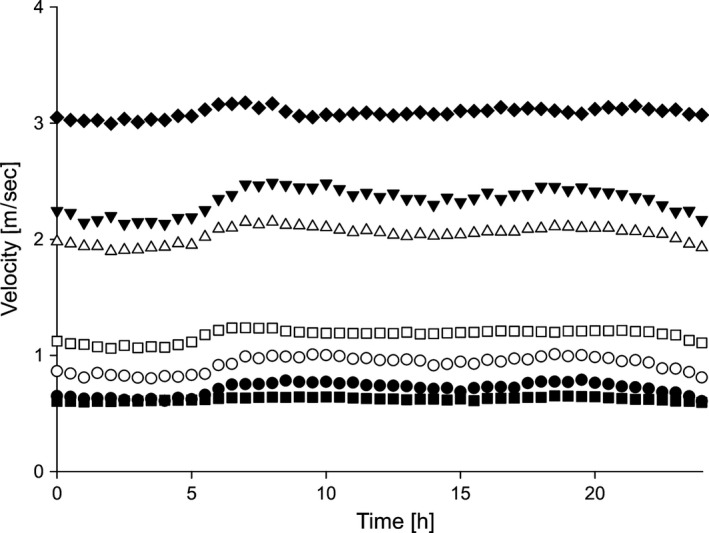
Average daily dry weather velocity profiles for the sampling sites (data from August through October). Locations: (●) FM001, (○) FM002, (▲) FM004, (△) FM007, (■) FM012, (□) FM017, and (♦) FM019.

The measured wastewater velocity from these sites was chosen as the parameter defining the sampling program as it can be related to the shear stress on the biofilms covering the submerged pipe wall in the sewer, with the assumption that the pipe geometry and wall roughness is fixed. Based on the dry weather data, the average daily velocity profile for each measuring station was calculated and is shown in Figure 2.

Based on the velocity profiles in Figure 2, sites FM004, FM012, and FM019 were chosen as sampling sites, FM012 represented a site with low velocity, and sites FM004 and FM019 represented sites with higher velocity.

### Sampling

Samples of wastewater and biofilms were collected by entering the manholes at the same time as the routine maintenance of the flow monitors, and the samples were collected during the day (usually around 10:00 am). Wastewater samples were collected in November 2009, March 2010, and June 2010. For each manhole, 1 L of wastewater was collected in triplicate at each time point in sterile glass bottles, and biofilm samples were collected in March 2010 and June 2010. The biofilm samples were collected using a biofilm sampler constructed from a sponge attached to a piece of PE plastic. These were sterilized by autoclaving prior to use and kept in sterile 50 mL falcon tubes. The biofilm samples were collected by scraping the sponge over the pipe wall immediately above the water line to avoid mixing with the wastewater. The samples were collected in the upstream sewer pipe discharging into the manhole rather than in the actual manhole. Triplicate biofilm samples were again collected at each location. The samples were transported to the laboratory within 12 h of sampling in a cool bag to ensure stable temperature following sampling.

### DNA extraction

Upon return to the laboratory, 50 mL samples of wastewater were filtered through a 0.22‐μm, 47 mm diameter, polycarbonate membrane filter (Millipore Ltd., Watford, UK). The filters were then stored at −20°C until the DNA was extracted. The biofilm samples were frozen at −20°C upon return to the laboratory. DNA was extracted using the MoBio Ultra Clean Soil DNA isolation kit (MoBio, Carlsbad, CA) following the manufacturer's instructions. DNA was extracted from half a filter for the wastewater samples, corresponding to 25 mL of the original 50 mL sample volume, and from a quarter of a sponge (1 cm^2^) for the biofilm samples and eluted in a final volume of 50 μL nuclease‐free water.

### Terminal restriction fragment length polymorphism (T‐RFLP) analysis

After DNA extraction, bacterial 16S rRNA genes were amplified using the polymerase chain reaction (PCR) with primers FAM63f and HEX1389r (Marchesi et al. 1998; Osborn et al. 2000). The primers were labeled at 5′ end of the DNA with the dyes 6‐FAM and HEX, respectively. The PCR was performed by using 1× PCR buffer, 1× Q solution (Qiagen, Crawley, UK), 200 μmol/L of each dNTPs, 0.3 μmol/L of both forward and reverse primers, and 2.5 U of Taq DNA polymerase (Qiagen). In each PCR, 2 μL of DNA extract was used as template and the final volume was adjusted to 50 μL using sterile nuclease‐free water. The DNA was amplified using an initial denaturation at 95°C for 5 min, followed by 35 cycles of 95°C for 30 sec, 57°C for 45 sec, and 72°C for 1 min. After the cycles, a final extension at 72°C for 10 min was applied. All PCR products were visualized by agarose gel electrophoresis (1% wt/v) and purified using QIAquick PCR purification kit (Qiagen) following the manufacturer's protocol. For the T‐RFLP analysis, 5 μL of purified PCR product from each DNA extract was digested with 20 U of Alu1 restriction enzyme (Roche, IN) along with 1.5 μL buffer supplied by the manufacturer and the final volume for each digestion reaction was adjusted to 15 μL using sterile nuclease‐free water. The digestion was performed at 37°C for 3 h after which the digested products were desalted using ethanol precipitation using glycogen at 20 mg/mL and 0.2 mmol/L magnesium sulfate heptahydrate. The desalted products were dissolved in 5 μL of sterile nuclease‐free water. The terminal restriction fragments (T‐RFs) were analyzed using an ABI 3730 DNA Analyzer (Applied Biosystems, Warrington, UK). The size of the T‐RFs was measured relative to a GS500 size standard (Applied Biosystems) and the size (nucleotides, unit of nt) was determined using GeneMapper^®^ software (version 3.7 Applied Biosystems, Foster City, Califormia, USA).

### Data analysis

The GS500 size standard ranged from fragments of between 35 and 500 bp. Therefore, all T‐RF's shorter than 35 bp and longer than 500 bp were removed from the profiles. The T‐RFLP profiles were then aligned using the T‐align tool (Smith et al. 2005). Data from T‐RFLP analysis are known to contain noise as well as the signal from the samples which means that a filter needs to be applied to remove the noise from the data (Osborn et al. 2000; Schütte et al. 2008). Schütte et al. (2008) recommended that the noise filter should be chosen in such a way that the correlation between the total number of peaks and the total area of the peaks was minimized. This is to ensure that any increase in observed taxon richness is not due to variations in the amount of DNA analyzed in the T‐RFLP analysis (Schütte et al. 2008). The minimization of this correlation was found to be achieved best by applying a percentage threshold to the data, hence for each profile it was calculated which percentage each peak contributed to the total area of the peaks (Sait et al. 2003). Peaks lower than a set threshold were then excluded from the dataset as noise. Thresholds of 0.5%, 1%, and 2% were tested, and a 1% threshold was chosen as the lowest threshold where the correlation between the number of peaks and area of the peaks was minimized.

After the application of the noise filter, the profiles were normalized so that the total area under each profile was one. Dendrograms were constructed from these data using the software “Primer‐E.” The similarity index between profiles was calculated using the Bray–Curtis similarity index. Statistically significant clusters were identified using a similarity profile (simprof) test with a 5% confidence level (Clarke et al. 2008).

## Results

Samples for bacterial community analysis were collected from three flow monitor locations in the catchment. The average dry weather wastewater velocities recorded during the sampling period was 2.3 m/sec (standard deviation 0.1 m/sec) for FM004, 0.6 m/sec (standard deviation 0.01 m/sec) for FM012, and 3.1 m/sec (standard deviation 0.05 m/sec) for FM019. These average velocities are based on dry weather data defined as described in Materials and Methods. The data for this analysis were collected between November 2009 and September 2010 and are based on an average of 100 dry weather days for each sampling point. For the T‐RFLP profiles, between 12 and 28 T‐RFs were detected in each sample after the 1% noise filter was applied and the average number of T‐RFs was 21 (standard deviation 4.7). All sampling was done on dry weather days. Prior to the sampling in November, it had rained the day before sampling. For the sampling in March, there had been 4 dry weather days prior to sampling and for the sampling in June there had been 6 dry weather days before the samples were collected.

### Difference between wastewater and biofilm communities

**Figure 3:**
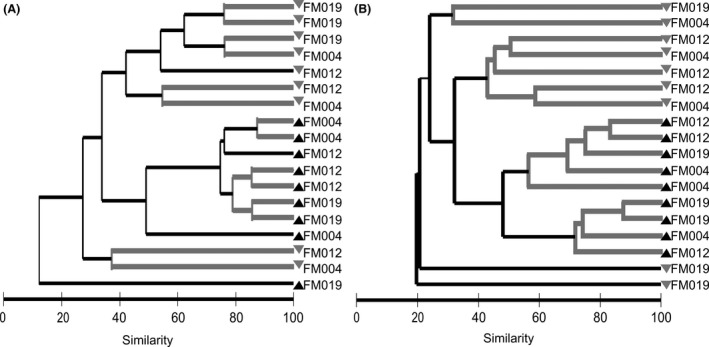
Dendrograms for the samples collected in March (A) and June (B) at the three sampling locations. Dendrograms were generated from a Bray–Curtis resemblance matrix derived from T‐RFLP analysis of PCR‐amplified 16S rRNA genes. Sample type: (▲) wastewater sample, (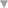) biofilm sample. Gray lines denote profile similarity below 95% significance based on the simprof test.

There were two sampling times where samples of wastewater and biofilms were collected at all three sites, that is, the March and June samples. Figure 3 shows the dendrograms displaying clustering of the bacterial communities. For the dendrograms in Figure 3, it is clear that the wastewater communities formed a distinct cluster from the biofilm communities in both March and June, with the exception of one outlier at FM019 for wastewater in March. In addition, greater variation was found between the biofilm communities and was observed in the wastewater communities which were more closely related to each other. Several samples from the same sampling location clustered together, particularly the wastewater samples, however, this trend was not consistent. There appeared to be no obvious effect of the level of the flow velocity upon the structure of the bacterial communities at each site for either the biofilms or wastewater samples.

**Figure 4:**
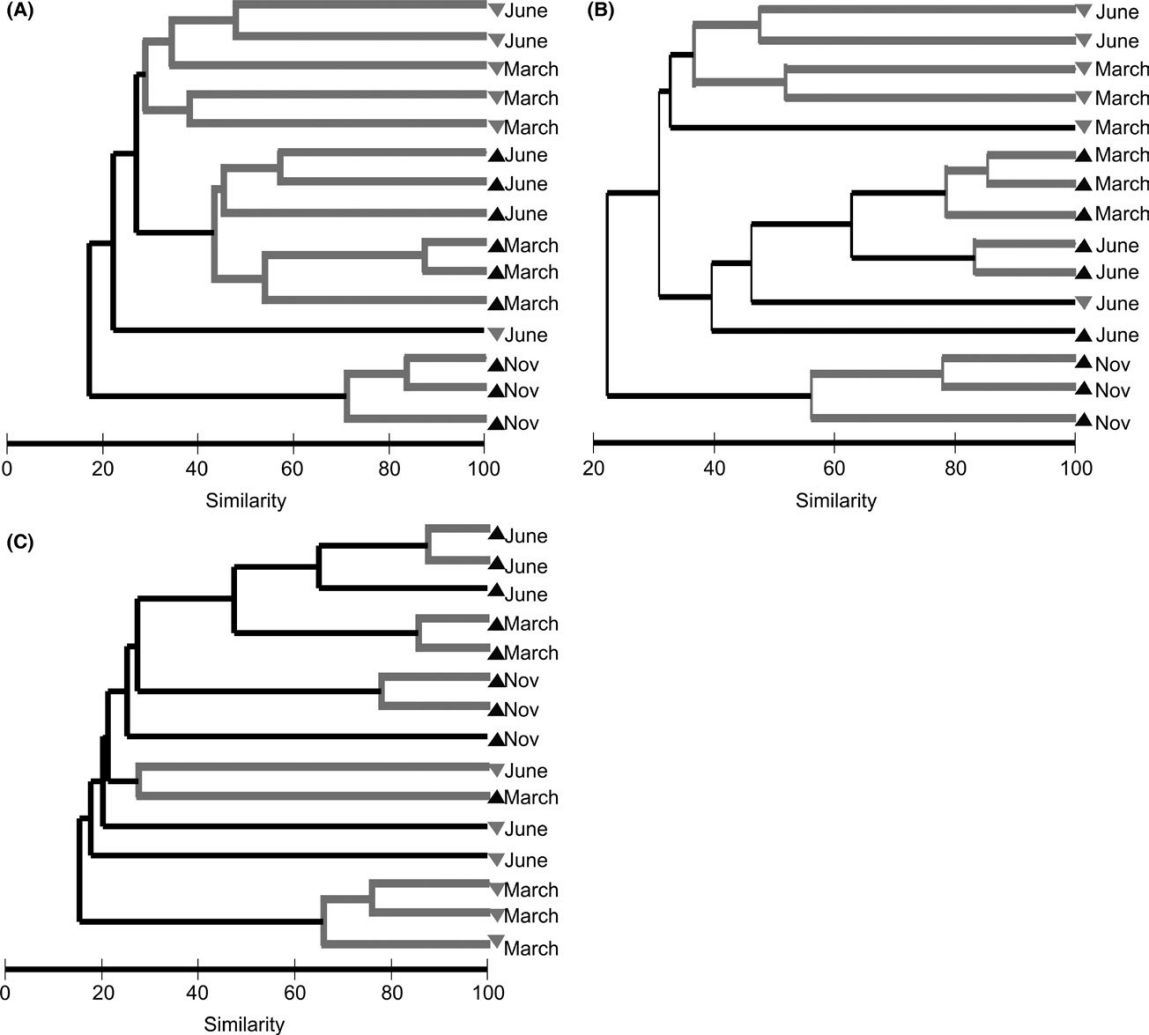
Dendrograms for the samples from the individual locations in the sewer network. Dendrograms were generated from a Bray–Curtis resemblance matrix derived from T‐RFLP analysis of PCR‐amplified 16S rRNA genes. (A) FM004, (B) FM012, and (C) FM019. Sample type: (▲) wastewater sample, (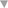) biofilm sample. Gray lines denote profile similarity below 95% significance based on the simprof test.

When comparing bacterial community structure between the three individual sampling locations in the network (Fig. 4), the wastewater and biofilm communities generally clustered separately from each other at each of the sampling locations. For example, both FM004 and FM012, the wastewater samples from November formed a distinct separate cluster. However, this was not the case for FM019, for which the biofilm communities in March formed a discrete cluster.

**Figure 5:**
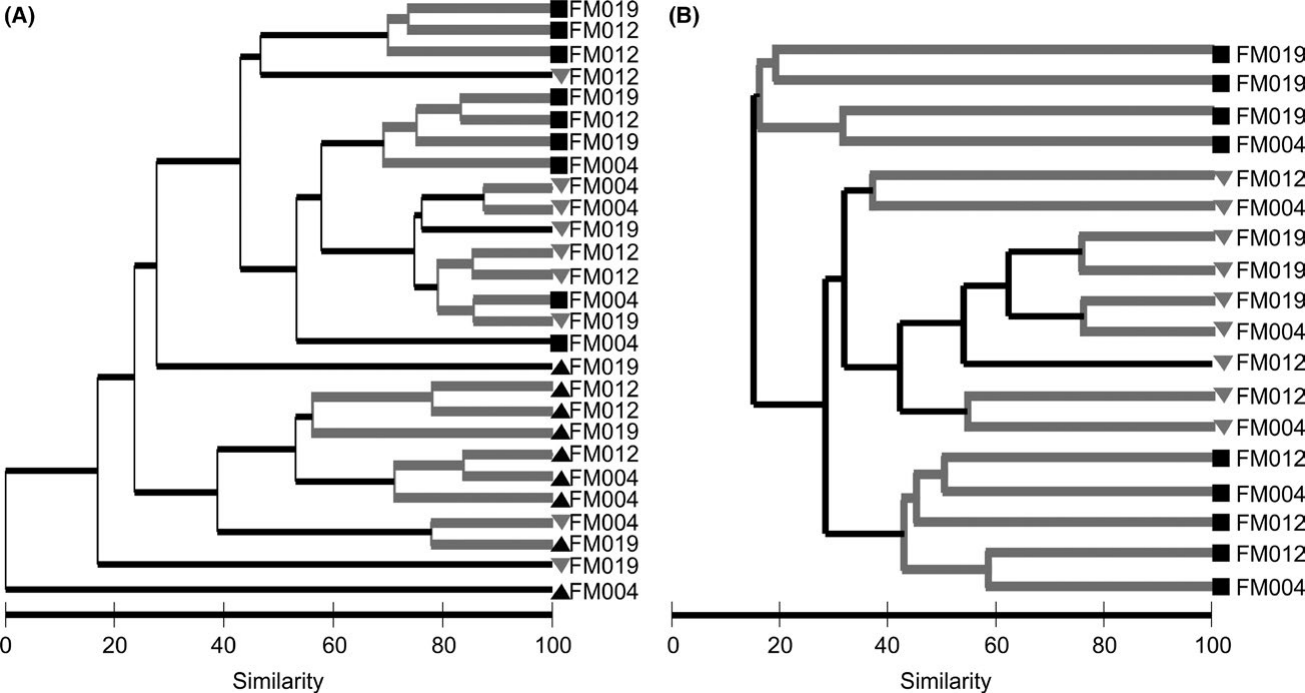
Dendrogram containing the similarities between all the (A) wastewater and (B) biofilm samples collected. Dendrograms were generated from a Bray–Curtis resemblance matrix derived from T‐RFLP analysis of PCR‐amplified 16S rRNA genes. (▲) denotes samples from November, (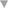) samples from March, and (■) samples from June.

Separating all of the biofilm communities from the wastewater communities shows that the biofilm communities overall formed three separate clusters (Fig. 5B) with separation of communities between month of sampling. One of the clusters consists of the biofilm communities from March, whereas the biofilm communities from June are divided into two clusters. The communities from FM012 and FM019 were generally separated by these two clusters, but the communities from FM004 are present in both clusters. Within the cluster of communities from March there is also a weak separation between FM012 and FM019, indicating that the differences between these two sites results in small, detectable differences in the biofilm community structure. However, it is clear that the effects between different sampling months are much larger than the impact of the local hydraulic conditions. The clustering for the wastewater communities was more dependent on the time of sampling than the sampling site.

### Flow data analysis

The catchment where the sampling was carried out is located in an area where infiltration of groundwater into the sewer networks is typically observed. Groundwater infiltration is a seasonal process, and is much more influential during certain months in which the local ground water levels are elevated due to previous rainfall (Kracht et al. 2008; Ellis and Bertrand‐Krajewski 2010). In order to determine if this is also the case at the sampled locations, a comparison was made between the average flow rates in the sewers in winter time – defined as the months from November through February (high infiltration possible) – in comparison to summer time, defined here as May through August. The flow rates used in this analysis are based on the data collected between November 2009 and September 2010 and the dry weather days were identified as described in Materials and Methods, the data for this analysis were collected between November 2009 and September 2010.

**Figure 6:**
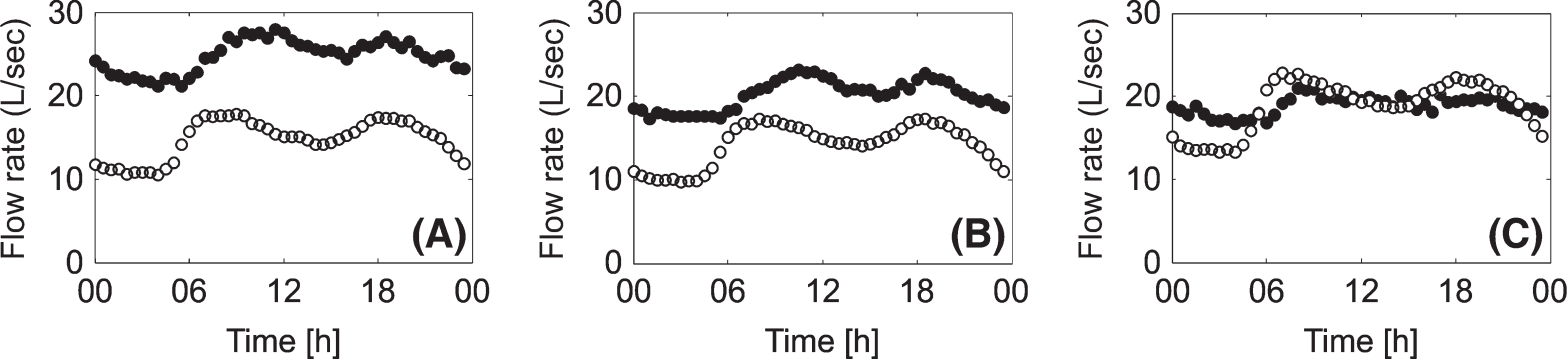
Average hydraulic daily dry weather flow rate profiles for (●) winter (November–February) and (○) summer (May–August) for the three sites, (A) FM004, (B) FM012, and (C) FM019.

Figure 6 shows that the average dry weather flow at each of the three sampling locations are higher during winter than in summer. Each curve is based on 28 dry weather days on average.

## Discussion and Conclusions

In this study, variations in the structure of bacterial communities in sewer wastewater were investigated at three locations along the same intercepting sewer and at three separate points in time. Variation in biofilm community structure was also investigated at the same locations, but at only two time points. In addition to the samples for microbial analysis, an extensive dataset of flow data and rain data were available for the catchment. From the bacterial community profiling it was clear that the biofilm communities were different from the wastewater communities at each sampling point and at each time (Figs. 3, 4). There are limited published studies comparing microbial biofilm communities with wastewater communities, so it is interesting that this distinction is quite clear, even if it is not unexpected. A lot of the understanding regarding the differences in biofilm and wastewater microbial communities in sewers comes from the investigation of in‐sewer processes, for example, aerobic heterotroph respiration and sulfate reduction. For aerobic oxidation of organic matter (heterotrophic respiration), it is generally accepted that the biofilm contributes significantly to these processes in the gravity sewers that this needs to be included in process models (Hvitved‐Jacobsen 2002). For sulfate respiration, it is generally accepted that the biofilm bacterial communities will be more influential on the process as the bacteria are slow growing relative to the normal residence time for wastewater in sewers. Significant variations in communities of sulfate‐reducing bacteria in biofilms alone have been found using DGGE methods along a rising main, showing a clear spatial variation over the 410 m pipe investigated (Mohanakrishnan et al. 2009). However, to the knowledge of the authors, similar studies using direct molecular microbial community analysis on both wastewater and biofilms have not been carried out for sewers. A study of water and biofilm communities around a hot spring in Argentina showed a clear distinction between the water and biofilms (Urbieta et al. 2015), it is, however, interesting that the difference between biofilm communities and bulk water communities is also clear in an environment as nutrient rich as the sewer environment.

Looking only at the wastewater communities in Figure 4, the samples taken in November cluster separately to those from March and June, which are grouped much closer (aside from one of the March samples). One reason for this may be that the November sewer flows are more influenced by infiltration of ground water into the sewer pipes (Fig. 6). Ground water infiltration alters the composition of the wastewater, and also reduces wastewater temperature along with cooler winter air temperatures within the sewer. Abdel‐Aal et al. (2014) measured seasonal temperature differences in wastewater and in‐sewer air temperatures of over 10°C in a Belgian combined sewer over a 12‐month period. Analyzing the average dry weather flow in winter compared to summer indicated that this particular system is influenced by infiltration as the winter flow is consistently higher than that of summer (Fig. 6). This may mean that the microbial communities in the wastewater in November may potentially be influenced by the natural communities in shallow ground water reservoirs. However, the infiltration generally also mean that the temperature in the wastewater is lowered and the concentration of organic matter is decreased (Karpf and Krebs 2011), which could also cause changes in the microbial communities in the sewer. When looking at all the biofilm communities together, there is clear variability between the two different sampling times (Fig. 5), with distinct groupings for March and June, whereas the groupings distinct for the individual sites are less clear. This indicates that even though local variability causes differences between the triplicate samples, the strongest variation in the biofilm communities are caused by time‐dependent variabilities in the overall sewer network. Temperature is one of the parameter that has been shown to cause changes to the metabolic activity of bacteria from sediments in gravity sewers (Biggs et al. 2011), and between March and June, the temperature in the sewer network is likely to have changed by an order of at least 10°C (Abdel‐Aal et al. 2014). Other studies have shown that there is a dynamic between the air temperature in the sewer and the wastewater temperature. During winter, the wastewater temperature is generally higher than that of the air in the sewer, around March and April, the wastewater and air temperature are similar, during summer the winter pattern is, when the air temperature will exceed that of the wastewater (Abdel‐Aal et al. 2014).

In addition to the general difference between the communities in the wastewater and the biofilms, the communities also indicate a higher variability overall in the biofilms than is found in the wastewater samples. This is particularly seen for samples collected at the same time in March and June (Fig. 3). The higher variability in the triplicate samples of biofilms compared to those for wastewater suggests greater spatial variability on the pipe surfaces for the biofilm communities compared to the slight temporal variability in the wastewater due to flow. Data from FM004 and FM012 clearly exhibit this higher variability between biofilm triplicates compared to variability between triplicates from the wastewater. However, at FM019 there is also some variability in the wastewater communities, as well as for the biofilm communities. The samples for biofilms in March and June were taken after 4 and 6 dry days prior to the sampling day, respectively. It is not clear if the number of antecedent dry days makes a difference with respect to development or stability of biofilms in the systems. However, with the varied conditions in combined sewers, it is unclear if stable or mature biofilms are ever found, with biofilm structure likely to be dynamic.

The variability in microbial communities in sewers is of interest mainly due to their influence on in‐sewer processes which contributes to problems such as odor generation in the urban environment or corrosion of the sewer pipe walls. In addition, the vision of enhancing the naturally occurring in‐sewer processes to provide safe in‐sewer treatment of wastewater without the need for large energy inputs at large end of pipes wastewater treatment plants (Hvitved‐Jacobsen 2002) requires understanding of the variable environment in the sewers. Vollertsen et al. (2005) described the statistical variation for some of parameters describing microbial transformation of organic matter. Few long‐term studies under field conditions of in‐sewer processes have been conducted, and there is scant information available on the long‐term variation in the microbial communities and their influence on the processes, beyond the direct metabolic effects of changing temperatures. This study has taken a first step to investigate the variability in bacterial communities directly in sewers. More work is needed to clarify how this relates to changes in in‐sewer processes and hence the influence on some of the problems caused by biochemical transformations in sewers.

## Conflict of Interest

None declared.
